# Challenges and Strategies for Prevention of Multidrug-Resistant Organism Transmission in Nursing Homes

**DOI:** 10.1007/s11908-017-0576-7

**Published:** 2017-04-05

**Authors:** Ghinwa Dumyati, Nimalie D. Stone, David A. Nace, Christopher J. Crnich, Robin L. P. Jump

**Affiliations:** 1 Infectious Diseases Division and Center for Community Health, University of Rochester, 46 Prince St, Rochester, NY 14607 USA; 2 Division of Healthcare Quality Promotion, Centers for Disease Control and Prevention, 1600 Clifton Road, Atlanta, GA 30329-4027 USA; 3 Division of Geriatric Medicine, Department of Medicine, School of Medicine, University of Pittsburgh, 3471 Fifth Ave, Kaufman Medical Building, Suite 500, Pittsburgh, PA 15213 USA; 4 University of Wisconsin, Madison, WI. Geriatric Research Education and Clinical Center (GRECC), William Middleton Memorial Veterans Hospital, 2500 Overlook Terrace, Madison, WI 53705 USA; 5 Case Western Reserve University, Cleveland, Ohio. GRECC, Louis Stokes Cleveland Veterans Affairs Medical Center, 10701 East Boulevard, Cleveland, OH 44106 USA

**Keywords:** Nursing home, Infection prevention and control, Methicillin-resistant *Staphylococcus aureus*, Multidrug-resistant organisms, *Clostridium difficile*

## Abstract

**Purpose of Review:**

Nursing home residents are at high risk for colonization and infection with bacterial pathogens that are multidrug-resistant organisms (MDROs). We discuss challenges and potential solutions to support implementing effective infection prevention and control practices in nursing homes.

**Recent Findings:**

Challenges include a paucity of evidence that addresses MDRO transmission during the care of nursing home residents, limited staff resources in nursing homes, insufficient infection prevention education in nursing homes, and perceptions by nursing home staff that isolation and contact precautions negatively influence the well being of their residents.

**Summary:**

A small number of studies provide evidence that specifically address these challenges. Their outcomes support a paradigm shift that moves infection prevention and control practices away from a pathogen-specific approach and toward one that focuses on resident risk factors.

## Introduction

Preventing healthcare-associated infections (HAI) and improving safety for residents in nursing home settings, which provide both post-acute skilled nursing and rehabilitation as well as long-term residential care, is a national priority [1]. Frail and older adults receiving care in nursing homes are at higher risk of infections due to age-related immune senescence, accumulation of comorbid conditions, and a higher burden of functional and cognitive deficits that increase dependence on caregivers. Additionally, the social and interactive nature of nursing homes such as shared dining, recreation, and therapeutic facilities generates opportunities for communicable diseases to spread.

The prevalence of risk factors for acquiring multidrug-resistant organisms (MDROs) and *Clostridium difficile* among nursing home residents has also increased with the growing complexity of medical care delivered in these settings. The post-acute, short stay population frequently requires invasive medical devices (e.g., urinary and vascular catheters) [2], wound management, and experiences more frequent healthcare and antibiotic exposure, all of which contribute to the emergence and spread of resistant pathogens. Here, we focus on recent evidence that supports integrating resident risk factors, such as the presence of indwelling catheters and gastrostomy tubes, into comprehensive infection prevention and control strategies to reduce MDRO transmission and acquisition.

## Prevalence of Healthcare-Associated Infections in Nursing Homes

Few studies have described the prevalence of HAI in nursing homes across the USA. Based on single facilities or clusters of facilities, Strausbaugh and Joseph estimated that 1.6 million to 3.8 million infections occur each year in the nursing homes, with a range of 1.8–13.5 infection per 1000 resident days [3]. A national point prevalence survey reported that in 2004, 12% of residents had an infection acquired either during a recent hospitalization or at a nursing home [4]. The Department of Veterans Affairs (VA) reported that in 2005 and 2007, the prevalence of infections among residents of VA nursing homes, termed community living centers (CLCs), was 5% [5]. Notably, for residents with indwelling devices, the rate was nearly 11%. Urinary catheters and gastrostomy tubes were the most common devices but more recently the prevalence of intravascular catheters has increased due to higher number of nursing homes providing post-acute infusion-related services [2]. A point prevalence study including several European countries reported that in 2013, 3.4% of nursing home residents had an HAI, with the prevalence rate in individual countries ranging from 0.4 to 7% [6].

## Infection, Colonization, and Risk Factors for Multidrug-Resistant Organisms and C. *difficile*

The rate of MDRO infections among nursing residents is not well described. Kahvecioglu et al. examined this using the Centers for Medicaid & Medicare Services Long Term Care Minimum Data Set (MDS), a standardized assessment tool that is implemented on admission to the nursing home and quarterly thereafter [7]. They found a rate of MDRO infections of 4.2% among nursing home residents across the USA, with a range from 1.9 to 11.4% among individual states.

Several studies describe that colonization with MDROs is common in the nursing home population. The rate of methicillin-resistant *Staphylococcus aureus* (MRSA) colonization among NH residents may exceed 50% [8, 9], far greater than the rate of 5–10% among hospitalized patients [10, 11]. While the prevalence of vancomycin-resistant *Enterococcus* (VRE) ranges from 4 to 16% [12, 13], the prevalence of multidrug-resistant Gram-negative bacilli is increasing [14]. Some studies found that approximately 50% of NH residents are colonized with quinolone-resistant Gram-negative bacilli compared to 17 and 1% colonization with extended-spectrum β-lactamase (ESBL)–producing and carbapenem-resistant *Enterobacteriaceae* (CRE), respectively [12, 13, 15–18].

Recent antibiotic exposure (within 4 months) correlated with an over fivefold increased risk of colonization with a drug-resistant Gram-negative bacilli [19] and with acquisition of MRSA [8, 20]. Additionally, dependence upon assistance with activities of daily living (ADLs) increased the risk of acquiring an MDRO. In a study of 82 nursing home residents, Fisch et al. found that most residents (53 to 71%) became colonized with MDROs after admission to the nursing home and reported a higher rate of functional dependence among those that became colonized compared to those that did not [12]. The time to acquisition varied based on the bacteria, ranging from an average of 186 days (±108 days) for VRE and 127 days (±79 days) for MRSA to just 76 days (±66 days) for ciprofloxacin-resistant Gram-negative bacilli. The median length of stay in long-term care facilities is 463 days [21]. Other risk factors for colonization with MDRO include the presence of indwelling medical devices, decubitus ulcers, presence of wounds, and urinary and fecal incontinence [8, 13, 22, 23].

In the last 10 years, colonization with *C*. *difficile* of NH residents ranged from 4 to 50% and a recent meta-analysis that included reports from Canada, Germany, and Ireland reported a pooled mean of 15% [24–26]. Compared with MDRO infections, more robust data is available for the rates and impact of *C*. *difficile* infection among nursing home residents. Campbell et al. determined the incidence rates in Ohio nursing homes to be 1.7–2.9 per 10,000 residents-days in 2006 [27], which is similar to the 2.3 per 10,000 residents-days (interquartile range [IQR], 1.2–3.3) rate reported in nursing homes in Monroe County, NY in 2010 [28]. Hunter et al. used a population-based surveillance approach to estimate that the rate of nursing home onset *C*. *difficile* infection in the USA for 2012 was over 110,000 cases [29]. Over 25% of those individuals required hospitalization, nearly 20% had a recurrence and 8% died. These data support the concern that *C*. *difficile* is endemic in some long-term care settings [30].

Recognized risk factors for *C*. *difficile* carriage among nursing home residents include a previous history of *C*. *difficile* infection, antibiotic use in the prior 3 months, and recent hospitalization [25, 26]. Furthermore, following treatment for *C*. *difficile* infection, residents continue to shed spores onto their skin where they may contaminate the hands of health care personnel [25, 31]. Finally, asymptomatic carriers also shed *C*. *difficile* spores into the environment for 1 to 4 weeks after therapy, where they may remain viable for at least 5 months on environmental surfaces [25, 32]. Both nursing home residents and their environment may serve as reservoirs for *C*. *difficile* spores.

## Nursing Homes as Regional Reservoirs for Multidrug-Resistant Organisms and C. *difficile*

The growing prevalence of MDROs in nursing homes has impact on regional efforts to prevent the spread of resistant pathogens. Kahvecioglu et al. found that among nursing home residents who became infected with an MDRO, 57% became positive in nursing homes and 41% in acute care settings. Such data supports consideration of regional approaches to infection prevention and control [7]. Two independent studies of the genetic heterogeneity of MRSA strains in regional nursing homes and hospitals reached similar conclusions [33•, 34]. Similarly, several epidemiological investigations of carbapenem-resistant Gram-negative bacteria also highlight the interconnectedness of acute care hospitals and post-acute and long-term care settings, including nursing homes. Perez et al. studied the epidemiology of carbapenem-resistant *Acinetobacter baumannii* and *Klebsiella pneumoniae* strains in Northeast Ohio, finding two predominant genotypes of each species among patients at three regional hospitals and one long-term acute care facility [35]. Similar case clusters of CRE occurred in West Virginia and in four adjacent counties in Indiana and Illinois with a single nursing home and long-term acute care facility, respectively, identified as playing an important role in transmission [36, 37]. More recently, serial surveillance cultures of residents with advanced dementia revealed transmission of multidrug-resistant Gram-negative bacteria among residents as well as between 22 nursing homes near Boston, Massachusetts [38•].

Just as the burden and transmission of an MDRO in one setting may affect regional healthcare, efforts to reduce MDRO colonization in one setting has the potential to reduce the risk of MDRO infections in other institutions. Simulation models based on data from Orange County, California suggest that improving infection prevention and control practices may have regional benefits. Lee et al. reported outcomes from a modeling study indicating that contact precautions for nursing home residents colonized for MRSA, with a 50% adherence rate, might reduce the prevalence of MRSA by 14% in nursing home settings and 2% in hospital settings [39]. Further modeling of universal MRSA decolonization of ICU residents projected a 3.0 and 1.9% reduction in the prevalence of MRSA colonization in long-term acute-care and nursing homes, respectively [40].

Overall, while the rates of new-onset *C*. *difficile* infection are higher for hospitalized patients, the larger number of nursing home beds means that the disease burden is greater among the long-term care population as a whole [27, 28]. The period of highest risk for infection is within 5 weeks of admission, likely reflecting vulnerability induced by antibiotic exposure during hospitalization coupled with exposure to spores in the nursing home [28, 29, 41].

## Challenges to Implementing MDRO Prevention in Nursing Homes

Several authoritative and academic health organizations have issued guidelines addressing infection prevention and control for MDROs in nursing homes [42–46]. The intent of the guidelines is to provide structure and function for establishing infection control programs in nursing homes. Studies in long-term care settings are limited and unfortunately, most of the recommendations are based primarily on evidence extrapolated from acute care settings. This discord highlights several challenges to infection prevention and control in nursing homes.

First, at the time guidelines on MDRO prevention and transmission-based precautions were written, there was a lack of evidence about effective infection prevention and control strategies in nursing homes. As described by Uchida et al. in a systemic review of randomized and non-randomized studies, the available evidence supporting infection prevention and control interventions in long-term care is limited, with a number of inconsistencies in implementation and significant knowledge gaps among staff [47]. In the USA, guidelines regarding isolation policies suggest that decisions regarding placing residents with MDRO colonization or infection on contact precautions should be made on a case-by-case basis [44]. Specific considerations include the impact on the social and psychological health of the residents as well as the risk for cross-transmission within the facility. Federal regulations require facilities to obtain permission from residents in order to move rooms, which favors the preferences of individual residents over the infection prevention and control practices that attempt to consider the safety of all residents. Ultimately, the decision to isolate, cohort or use contact precautions for a resident is left up to the nursing home staff, which is influenced by the availability of private rooms and the preference of residents and their families. Furthermore, most of the evidence nursing homes might use to guide those decisions is adapted from infection prevention and control strategies developed and validated in hospitals.

Second, compared to acute care facilities which are replete with on-site physicians, nurses, pharmacists, and robust infrastructure to support diagnostic testing, nursing homes are resource-limited settings. Very few nursing homes have in-house laboratories and most must contract with outside vendors for laboratory and radiology services. This often means that nursing homes cannot make use of advanced microbiological and serological tests due to limited access or prohibitive costs. Even the simple act of obtaining a chest radiograph or routine laboratory tests takes longer in nursing home settings and may sometimes require transfer to an emergency department. Accordingly, nursing home providers face diagnostic uncertainty due to lack of onsite medical providers and delays in the availability of test results. These factors, often combined with difficulty in communication of symptoms by residents with cognitive or hearing impairment, all contribute to overtreatment with antibiotics, which in turn increases the risk of colonization with MDROs and *C*. *difficile* infection [11, 12]. Furthermore, most nursing homes do not perform active surveillance cultures, which can be cost-prohibitive [48]. Rather, they identify residents colonized with an MDRO based on clinical cultures or based upon communications about MDRO history from the transferring facility during care transitions, which may be limited, inaccurate, or nonexistent. This practice, while understandable, likely underestimates the true prevalence of MDROs among nursing home residents [15, 49–51].

A third challenge is that nursing homes may not allocate sufficient personnel resources or education to support effective infection prevention and control, despite increasing numbers of more medically complex residents with greater risk for MDRO acquisition. Several studies reported that the role of infection preventionist is often filled by a nurse with both inadequate training and time to carry out those duties. In their national survey, Ye et al. found that only 25% of nursing homes had a full-time infection control professional [48]. A national survey conducted by Herzig et al. reported that over 50% of the people responsible for infection prevention and control had at least two other roles and over 60% had no specific training for this role [52]. Additionally, instability in nursing home staffing may influence the prevalence of MDROs, and thus the risk of residents becoming colonized. Certified nursing assistants (CNAs), who comprise the bulk of front line staff, perform a variety of tasks involving direct resident care which, if not performed correctly, may increase the risk of transmitting MDROs [53]. High rates of CNA turnover correlate with increased risk of several adverse outcomes. Furthermore, nursing homes often rely upon a mix of permanent employees as well as temporary personnel employed through staffing agencies. The transient nature of agency employees also undermines efforts to educate healthcare workers about infection prevention and control [53]. Fortunately, strategies to reduce staff turnover and improve education can be effective, as demonstrated in a study of VA CLCs where the risk of infection among residents decreased as the tenure of nurses increased [54]. Furthermore, nursing homes in states that require more than the federal minimum for clinical education for CNAs (16 h annually) had improved resident outcomes compare to those that did not [55].

The final challenge is cultural and rooted in the perceptions of nursing home staff about the influence of isolation and contact precautions on the welfare of their residents. The overarching goal of nursing homes is to offer supportive care and restorative services in an environment that respects their residents’ dignity, wellness, and quality of life. The use of the term *residents*, rather than *patients*, communicates that nursing homes are culturally distinct from acute care. The care of a resident colonized or infected with an MDRO creates a strain as nursing homes must consider the safety of other residents by minimizing their exposure to potential pathogens [56]. Furuno et al. explored the opinions of nursing home staff about isolation precautions for nursing home residents with MRSA or VRE [57]. They found that while staff understood that infection prevention and control practices reduce the risk of transmission of potential pathogens, they were also concerned about the adverse effects of these practices on individual residents, particularly depression. Extending these findings, Cohen et al. found in their study of ten nursing homes that staff expressed strong concerns about the influence of infection prevention and control practices on residents; one interviewee described isolation as “almost like holding a person prisoner” [58••]. Efforts to improve infection prevention and control practices in nursing homes will need to account for the concerns and perceptions of the front line staff ultimately responsible for their day-to-day implementation.

## Addressing the Challenges of MDRO Prevention and Control in Nursing Homes

Many of the MDRO prevention challenges identified above are driven by knowledge and resource gaps in the nursing home setting. Fortunately, a small but growing number of studies provide evidence that specifically addresses these challenges in nursing homes and other long-term care settings. An emerging theme from these studies is a shift in MDRO prevention strategies away from an approach based on the knowledge of a resident harboring a specific pathogen and toward an approach based on knowledge of resident risk factors for MDRO transmission and acquisition, such as the uncontained bodily fluids and indwelling devices.

First, given the evidence of cross-contamination between nursing home residents and caregivers, broad approaches to reduce the reservoir for MDRO transmission might have applications in the nursing home setting. A study by Roghmann et al. quantified the risk of MRSA contamination on the hands and clothes of healthcare personnel during interactions with MRSA-colonized residents [59••]. Rates of transmission increased from residents with chronic skin breakdown and, notably, during assistance with high-risk activities such dressing, transferring, providing hygiene, changing linens, and toileting (OR >1.0; *P* < .05). This study confirms the risk of MDRO transmission during common caregiving activities. Chlorhexidine bathing may be a potential strategy to help reduce transmission by decreasing the prevalence of MDROs among nursing home residents. At four long-term acute care facilities in Chicago, incorporating daily chlorhexidine bathing used as part of a bundled intervention led to a significant reduction of KPC colonization and infection [60•]. The bundled intervention included rectal screening patients for colonization with KPC, contact isolation, and geographic separation of KPC-positive patients in ward cohorts or single rooms and healthcare worker education and adherence monitoring. A similar approach may be successful in nursing homes, as suggested by a pilot study that used chlorhexidine bathing and nasal iodophor [61]. During the 3-month intervention, the three participating nursing homes achieved a decrease in the prevalence of MRSA (29 to 19%), VRE (12 to 4%), and ESBL (15 to 9%).

Second, potential strategies to address the limited resources in nursing homes may come from leveraging community and regional resources. The interconnectedness between the acute healthcare facilities and nursing homes suggests that coordinated approaches across care settings may be needed to reduce the burden of MDROs [62]. At a regional level, creating a multidisciplinary task force can facilitate the standardization of infection prevention and control guidelines, improve inter-facility communication about MDROs during care transitions [63], and provide infection prevention and control expertise. The Centers for Disease Control and Prevention (CDC) recently recommended a coordinated approach for the prevention of MDRO and *C*. *difficile* with the involvement of health departments, acute care hospitals, and post-acute and long-term care settings [64]. While specific recommendations and models for this type of multi-institutional, cooperative infection prevention and control effort are not yet available, two examples come from the control of VRE in the Siouxland region of Iowa, Nebraska and South Dakota and the control of CRE in Israel healthcare facilities [65, 66]. Both prevention efforts convened a task force comprising public health workers and personnel from acute and long-term care facilities. The task force generated guidelines for screening and infection control and also worked to improve communication among facilities. In the Siouxland region, the prevalence of VRE decreased from 2.2% in 1997 to 0.5% in 1999 among 30 acute and long-term facilities [65]. In Israel, the task force issued evidence-based guidelines in 2007 that were specific to long-term care facilities. The guidelines supported the care of CRE-colonized residents without compromising their ability to participate in rehabilitation and socialization important to their well-being [66]. Furthermore, in 2014, the Illinois Department of Public Health launched a statewide registry of extensively drug-resistant organisms which permits bidirectional, web-based exchange of information among acute care, long-term acute care, and long-term facilities. This approach supports regional infection prevention and control efforts and may overcome communication gaps that occur during care transitions [67, 68].

The third challenge is that of dedicating personnel and education to infection prevention and control. As part of a larger study, Koo et al. reported that didactic and interactive educational sessions for nurses, CNAs, and environmental service staff, repeated every 2–3 months over 3 years, improved staff’s infection prevention and control knowledge [69]. Differences in knowledge scores were noted between nursing and CNAs highlighting the need to tailor the education for people in different roles. Unfortunately, as predicted by behavioral health theory [70, 71], education alone is not sufficient to generate the sustained behavioral changes necessary to reduce the risk of MDRO acquisition among nursing home residents [72, 73]. Ho et al. described sharing a written infection control plan with nursing home staff for activities related to enteral feeds which successfully reduced the prevalence of MRSA [74]. Further studies might investigate the correlation between hours allocated to infection prevention and control activities, the training of the personnel performing those activities, and the prevalence of MDROs among nursing home residents.

Addressing the final challenge, which is the stigmatizing influence of isolation and contact precautions on the welfare of nursing home residents, may require a paradigm shift. Currently, identification of an MDRO prompts initiation of isolation precautions. Rather than implemented as a reaction to a specific communicable disease, transmission-based precautions could be applied proactively to nursing home residents based on their individual risk factors. This has the potential to reduce the social discomfort associated with having an MDRO as well as reduce the risk of MDRO transmission. In 2010, the California Department of Public Health introduced enhanced standard precautions for long-term care facilities. Specifically, any contact with a resident with uncontrolled or uncontained secretions or body fluids warrants using personal protective equipment (e.g., gloves, gowns, surgical masks, and eye protection) [75]. Mody et al. studied a similar approach, focusing on nursing home residents with indwelling devices. They used a cluster randomized trial to examine whether a multimodal-targeted infection program would reduce the prevalence of MDROs and incident device-related infections [76••]. The intervention included preemptive barrier precautions, active surveillance for several MDROs, and infections with feedback about those data as well as comprehensive infection prevention education to nursing home staff. For the barrier precautions, the nursing home instructed staff to wear gowns and gloves during the care of residents with indwelling devices such as feeding tubes and urinary catheters. The residents socialized and participated in rehabilitation outside of their rooms without additional precautions. The results showed a 23% reduction in the prevalence density of MDROs, which included MRSA, VRE, and Gram-negative bacteria resistant to either ciprofloxacin or ceftazidime (rate ratio 0.77, 95% CI 0.62–0.94).

While the above studies provide new paradigms for MDRO prevention in nursing homes, additional evaluations may be needed to address issues such as cost and resource allocation. For example, using a prospective, observational approach, Roghmann et al. estimated that for community nursing homes, the average cost of standard precautions was $100 per resident per month, $125 per resident per month if gowns/gloves were used for all residents with chronic skin breakdown, but increased to $223 if gowns and gloves were used for all high-risk care activities [77•]. The cost-analysis highlights the importance of providing data that can help nursing homes estimate, and thus prepare for, the costs related to new approaches to MDRO prevention and control.

## Conclusions

Age, comorbid illnesses, invasive medical devices, frequent antibiotic exposure, and dependence on healthcare workers, in the setting of communal living, all serve to increase the risk of nursing home residents becoming colonized or infected with healthcare-acquired bacterial pathogens*.* As more medically complex patients seek care, nursing homes need to adapt and improve their infection prevention and control approach and do so in a matter that facilitates staff adherence. Basing barrier precautions on the presence of an indwelling device or specific task, such as assistance with bathing, is much easier for all staff members to understand and incorporate into their workflow. Additionally, this approach may help decentralize the responsibility for infection prevention and control from a single individual to several staff members. Over time, a paradigm shift to an approach based on resident risk factors may lead to noticeable improvements in infection prevention and control that also upholds the quality of life, comfort, and dignity of older adults living in nursing homes. In addition to improving specific nursing home infection control and prevention programs, the development of collaborative efforts across local and regional healthcare facilities will enhance the overall success of reducing the burden of MDROs across the continuum of care.
